# Deposition of callose in young ovules of two *Taraxacum* species varying in the mode of reproduction

**DOI:** 10.1007/s00709-014-0654-8

**Published:** 2014-06-18

**Authors:** Krystyna Musiał, Maria Kościńska-Pająk, Renata Antolec, Andrzej J. Joachimiak

**Affiliations:** Department of Plant Cytology and Embryology, Institute of Botany, Jagiellonian University in Krakow, Gronostajowa 9, 30-387 Cracow, Poland

**Keywords:** Apomixis, Callose, Chromosome number, Diplospory megasporogenesis, *Taraxacum*

## Abstract

Although callose occurs during megasporogenesis in most flowering plants, the knowledge about its general function and the mechanisms by which the callose layer is formed in particular places is still not sufficient. The results of previous studies suggest a total lack of callose in the ovules of diplosporous plants in which meiosis is omitted or disturbed. This report is the first documentation of callose events in dandelions ovules. We demonstrated the pattern of callose deposition during the formation of megaspores through diplospory of *Taraxacum* type and during normal meiotic megasporogenesis in apomictic triploid *Taraxacum atricapillum* and amphimictic diploid *Taraxacum linearisquameum*. We found the presence of callose in the megasporocyte wall of both diplosporous and sexual dandelions. However, in a diplosporous dandelion, callose predominated at the micropylar pole of megaspore mother cell (MMC) which may be correlated with abnormal asynaptic meiosis and may indicate diplospory of the *Taraxacum* type. After meiotic division, callose is mainly deposited in the walls between megaspores in tetrads and in diplodyads. In subsequent stages, callose gradually disappears around the chalazal functional megaspore. However, some variations in the pattern of callose deposition within tetrad may reflect variable positioning of the functional megaspore (FM) observed in the ovules of *T. linearisquameum*.

## Introduction

Callose (β-1,3-glucan polymer) plays a significant role during reproductive processes occurring in the anthers and ovules of flowering plants. In the course of angiosperms sporogenesis, callose accumulates in the walls of cells undergoing meiotic division (Lersten 2004). It appears in the sporocyte wall at the early stage of meiotic prophase I and disappears at the tetrad stage. Callose not only temporarily isolates individual sporocytes but it also isolates male and female meiocytes as well as young spores from the influence of the surrounding sporophytic tissues, which is related to the shift from sporophytic to gametophytic gene expression. It has been proposed that callose functions as a molecular filter between the genetically different cells, decreasing the permeability of the cell wall, and it serves as a selective barrier which transmits only specific signals that are essential to meiosis (Heslop-Harrison and Mackenzie 1967; Rodkiewicz 1970; Bhandari 1984; Bouman 1984). The deposition of callose in the megaspore mother cell (MMC) wall is a specific feature of angiosperms with the monosporic and bisporic type of female gametophyte development, whereas callose is absent in species with the tetrasporic type of megagametophyte formation (Rodkiewicz 1970). This author also reported that the callose deposition pattern is apparently dependent on the mode of female gametophyte formation, and it is quite different in the species with *Polygonum* and *Oenothera* types of embryo sacs. Callose deposition has also been examined in the ovules of apomictic plants, and it should be emphasized that the patterns of callose accumulation in the ovules of sexual species are not preserved in the ovules of apomicts. Generally, diplosporous species are characterized by a total absence of callose deposition around MMCs, as has been demonstrated in *Elymus rectisetus* (Carman et al. 1991), *Poa nemoralis* and *P. palustris* (Naumova et al. 1993; 1999), *Tripsacum* species (Leblanc et al. 1993, 1995), *Eragrostis curvula* (Peel et al. 1997) as well as in the apomeiotic mutants of *Medicago sativa* subsp. *falcata* (Barcaccia et al. 1996), the meiotic mutants of *Zea mays* (Abramova et al. 2003), and in the some monosomic addition line of *Beta corolliflora* (Shen et al. 2006) in which the MMCs form embryo sacs by mitosis. Lack of callose has also been found in the cell walls of aposporous initial cells in aposporous *Poa pratensis* (Naumova et al. 1993), *Panicum maximum* (Naumova and Willemse 1995), *Pennisetum* (Peel et al. 1997), *Brachiaria decumbens* (Dusi and Willemse 1999), *Hieracium* (Tucker et al. 2001), *Hypericum perforatum* (Galla et al. 2011), and *Eulaliopsis binata* (Li et al. 2011), whereas in the same species, the accumulation of callose has been noticed in the walls of MMCs. In the case of diplosporous plants, the callose events have mainly been examined in the ovules of species exhibiting an *Antennaria* type of diplospory in which female meiosis is omitted (mitotic diplospory). Nevertheless, a reduction or complete absence of callose in MMC walls was also suggested in apomicts exhibiting meiotic diplospory of *Taraxacum* and *Ixeris* types (Carman et al. 1991; Peel et al 1997). In these types of diplospory, unreduced megagametophytes are formed from megaspores that are a result of a restitutional meiosis, i.e., a modified megasporogenesis without the first reduction division (Gustafsson 1946; Nogler 1984; Asker and Jerling 1992).

*Taraxacum* Wigg. (Asteraceae, Cichorioideae) is a very large genus which forms polyploidy complex comprising rare diploids and widespread polyploid taxa (Kirschner and Štěpánek 1996). Within the genus, as in other agamic complexes, sexuality is linked to diploid species, whereas polyploid dandelions usually reproduce asexually through apomixis (Richards 1973; Mogie and Ford 1988). In sexual dandelions, seed formation depends on meiosis and double fertilization, whereas apomicts produce viable seeds without both meiosis and fertilization and their reproduction involves three independent processes: (1) meiotic diplospory, (2) parthenogenesis, and (3) autonomous endosperm formation (Gustafsson 1946; Nogler 1984; Asker and Jerling 1992; van Dijk and Bakx-Schotman 2004).

To the best of our knowledge, callose events in the ovules have never been studied in the genus *Taraxacum*. Here, we document the course of megaspores formation in two dandelions: (i) *Taraxacum atricapillum* Sonck, which has also been the object of a karyological analysis carried out for the first time in this species and (ii) diploid *Taraxacum linearisquameum* Soest (2n=2x=16) (Góralski et al. 2009; http://www.binoz.uj.edu.pl). Moreover, the present study represents a comparison of callose deposition pattern during diplosporous and meiotic megasporogenesis in *T. atricapillum* and *T. linearisquameum*, respectively.

## Material and methods

### Plant material

Dandelion capitula at early developmental stages were used in this study. Inflorescences of *T. atricapillum* were collected from plants randomly taken from a natural population in Żabokliki (52° 11′ 05″ N, 22° 19′ 05″ E). For karyological analysis of this species, mature seeds were also sampled. Capitula of *T. linearisquameum* were collected from specimens growing in the private collection of Dr. Jolanta Marciniuk in Siedlce (52° 10′ 49″ N, 22° 18′ 26″ E); these plants were obtained from seeds collected by dr. R. Vašut in Moravian Silesia in the Czech Republic.

**Table 1:** Developmental stages in the analyzed ovules of *Taraxacum linearisquameum* and *T. atricapillum*

Developmental stages	Number of observed ovules			
	*T. linearisquameum* 2n=2x=16		*T. atricapillum* 2n=3x=24	
	Cleared	DAB staining	Cleared	DAB staining
Single archesporial cell	12	17	19	23
Two archesporial cells	–	–	6	–
Megaspore mother cell	16	27	33	25
Restitution nucleus	–	–	3	–
Dyad	9	21	–	–
Diplodyad	–	–	54	51
Triad	5	–	4	2
Tetrad of megaspores just after meiosis	20	28	–	–
Increased megaspores				
Chalazal	26	28	–	–
Subchalazal	7	6	–	–
Chalazal and subchalazal	-	2	–	–
Micropylar	8	9	–	–
Submicropylar	4	-	–	–
Micropylar and submicropylar	4	3	–	–
Micropylar and chalazal	2	5	–	–
Submicropylar and subchalazal	3	–	–	–
Total	116	146	119	101

### Karyological analysis

The procedure of preparing samples for chromosome counts was described earlier by Marciniuk et al. (2012). Seeds of *T. atricapillum* were germinated in Petri dishes. Four-day-old seedlings were incubated in 8-hydroxychinoline for 4 h at room temperature and then fixed in glacial acetic acid:96 % ethanol (1:3, v/v) for 24 h. Fixed samples were stained in 2 % acetic orcein for 3 days at room temperature. Stained material was transferred into 45 % acetic acid and heated to a boiling point over a flame. Then the root tip meristems were cut off and squashed in a drop of 45 % acetic acid, dry-iced, air-dried, and mounted in Entellan. The chromosomes were counted during the mitotic metaphase.

### Tissue clearing technique

Whole capitula of both *Taraxacum* species were fixed in glacial acetic acid:96 % ethanol (1:3, v/v) for at least 24 h and stored in 70 % ethanol. Individual flowers were then isolated and dehydrated for 30 min in 80 %, 90 % (one change), and 100 % ethanol (two changes). After dehydration, flowers were cleared in methyl salicylate using a modified procedure earlier described by Musiał et al. (2013) and Płachno et al. (2014). Samples were incubated in absolute ethanol/methyl salicylate solutions (3:1, 1:1 and 1:3, v/v) and in two changes of pure methyl salicylate (1 h per step). Cleared flowers were mounted under cover slip in a drop of pure methyl salicylate and examined using a Nikon Eclipse 80i microscope fitted with Nomarski interference contrast optics. A total of 235 ovules were analysed; 116 ovules of T. linearisquameum and 119 ovules of T. atricapillum (Table 1).

### Detection of callose

Decolorized aniline blue (DAB; 0.1 % *w*/*v*) was used to detect the presence of callose in the ovules, as described by Martin (1959). Individual flowers were dissected from fixed capitula, transferred to 80 % ethanol for 30 min, pretreated with 1 N NaOH for 4 h at 37 °C, and after three washes with distilled water and one with 0.1 M K_3_PO_4_, the softened samples were stained overnight in 0.1 % DAB in 0.1 M K_3_PO_4_ at room temperature. Then the flowers were placed into a drop of 0.1 M K_3_PO_4_:glycerol (1:1, v/v) on a microscope slide and ovules were dissected under a stereomicroscope. After ovule isolation, samples were gently squashed under a cover slip and observed under UV light using a Nikon Eclipse E400 microscope with an Epi-Fl Filter Block N UV-2A consisting of excitation filter EX330–380, dichroic mirror DM400, and barrier filter BA420. A total of 247 ovules were analyzed; 146 ovules of T. linearisquameum and 101 ovules of T. atricapillum (Table 1).

## Results

### Chromosome number in *T. atricapillum*

To date, *T. atricapillum* has not been karyologically investigated, and this is the first information on the chromosome number for this dandelion species. The chromosome count showed a triploid chromosome number 2n=3x=24.

**Fig. 1:**
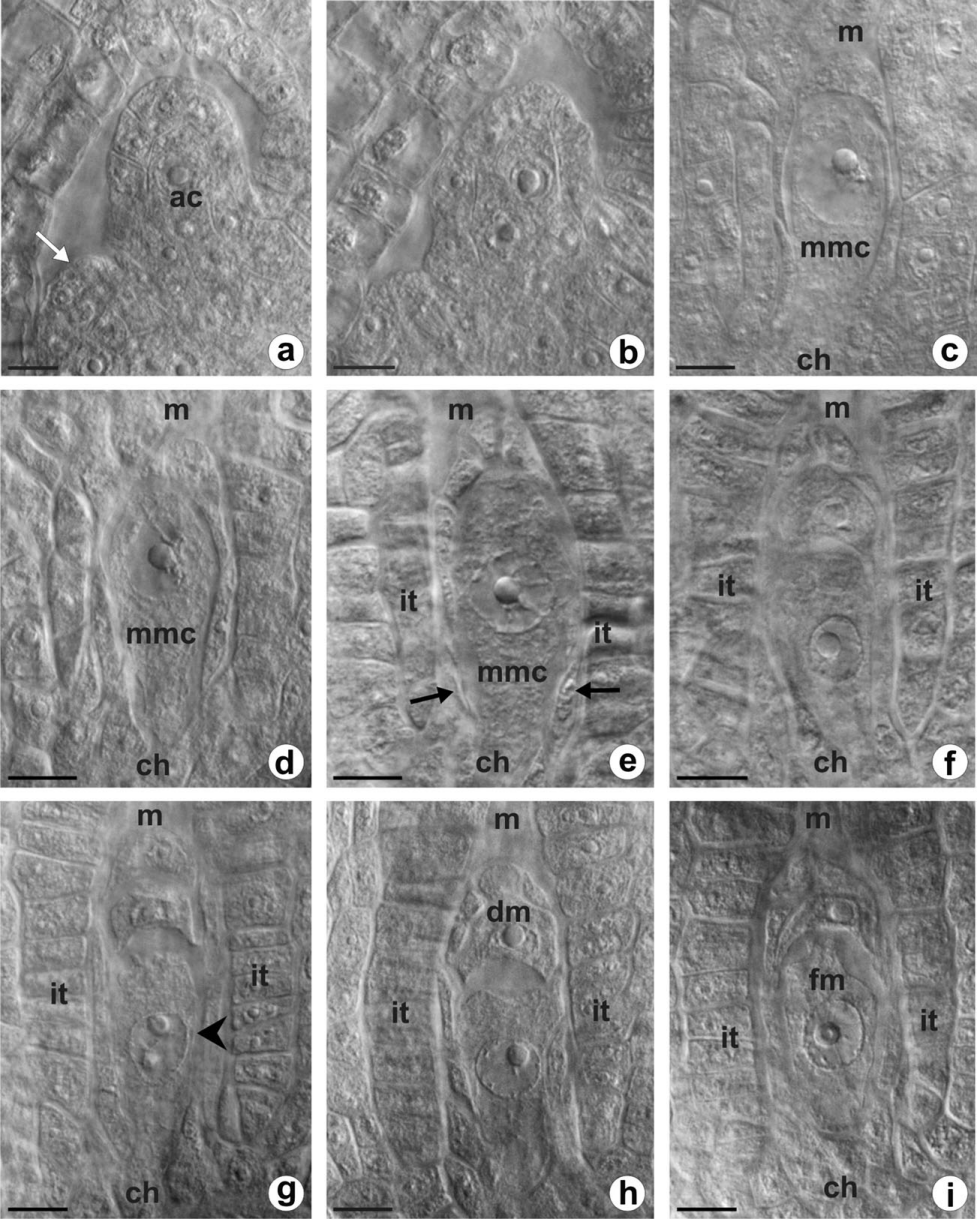
Megaspore formation in triploid *T. atricapillum*. Images were obtained from unstained, cleared flowers using Nomarski DIC optics. **a** Ovule primordium with a single archesporial cell (*ac*) visible in the hypodermal region of the nucellus; *arrow* indicates site of integument formation. **b** Two archesporial cells in young ovule. **c** Differentiated megaspore mother cell (*mmc*). **d**, **e** Prophase in megaspore mother cell (*mmc*); *arrows* point to crushed cells of nucellus. **f** Dyad of unreduced cells. **g** Triad of megaspore cells; increased chalazal cell points by *arrowhead*. **h** Diplodyad with enlarged chalazal megaspore and degenerating micropylar one (*dm*). **i** Functional megaspore (*fm*). *ch* chalazal pole, *it* integumentary tapetum, *m* micropylar pole. *Scale bars* = 10 μm

### Ovule development and megaspores formation in *T. atricapillum*

The dandelion ovule is unitegmic, tenuinucellate, and anatropous at maturity. A single ovule primordium develops as a dome-shaped protuberance of placental cells in an unilocular ovary (Fig. 1a, b). Formation of the integument begins at the base of a several-celled ovule primordium and coincides temporally with differentiation of the archesporial cell in the hypodermal region of the nucellus. During early ovule development, a single cell situated at the distal end of the ovule primordium, just beneath the epidermal cell layer, increases in size and displays a centrally positioned prominent spherical nucleus with a large nucleolus (Fig. 1a). Uncommonly, two archesporial cells are distinguished in the nucellus of young ovules of *T. atricapillum* (Fig. 1b). During further development, the ovule gradually curves, the integument overgrows the nucellus, and the archesporial cell becomes distinctly elongated in the micropylar-chalazal axis. The archesporial cell without intermediate division functions directly as the MMC, showing polarity with a prominent nucleus situated on the micropylar side (Fig. 1c). When the MMC enters into the first meiotic prophase, the nucellus remains uniseriate and is crushed between the integument and megasporocyte; however, the nucellus gradually degenerates and the cells of the innermost layer of integument differentiate into the integumentary tapetum adjacent to the MMC (Fig. 1d, e). In triploid *T. atricapillum*, the first meiotic division of MMC is altered and leads to the formation of a restitution nucleus. The second meiotic division is undisturbed and results in two unreduced megaspores, i.e., diplodyad (Fig. 1f). Only in four of the ovules analyzed at this stage, three megaspore cells unequal in size were observed as a result of abnormal meiosis (Fig. 1g). The micropylar cell of diplodyad degenerates, whereas the chalazal one increases in size and a small vacuoles within it begin to coalesce (Fig. 1h, i). This cell is a functional megaspore (FM) which undergoes three successive mitotic divisions leading to the formation of an unreduced female gametophyte. The megagametophyte develops similarly to the *Polygonum* type, and the organization of a mature unreduced female gametophyte is the same as that of a meiotic embryo sac.

**Fig. 2:**
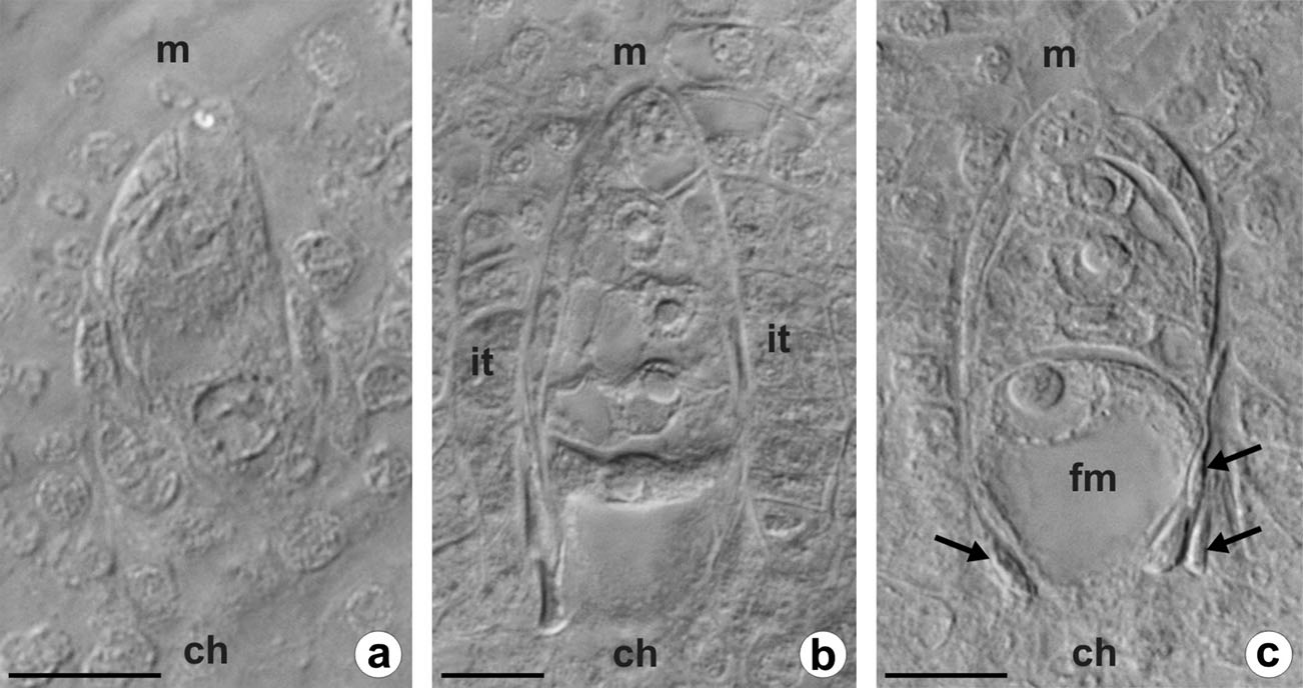
Megasporogenesis in diploid *T. linearisquameum*. Images were obtained from unstained, cleared flowers using Nomarski DIC optics. **a** Dyad of cells resulting from first meiotic division. **b** Young linear tetrad of megaspores. **c** Tetrad of megaspores with enlarged chalazal functional megaspore (*fm*); *arrows* point to crushed nucellar cells. *ch* chalazal pole, *it* integumentary tapetum, *m* micropylar pole. *Scale bars* = 10 μm

### Megasporogenesis in diploid *T. linearisquameum*

**Fig. 3:**
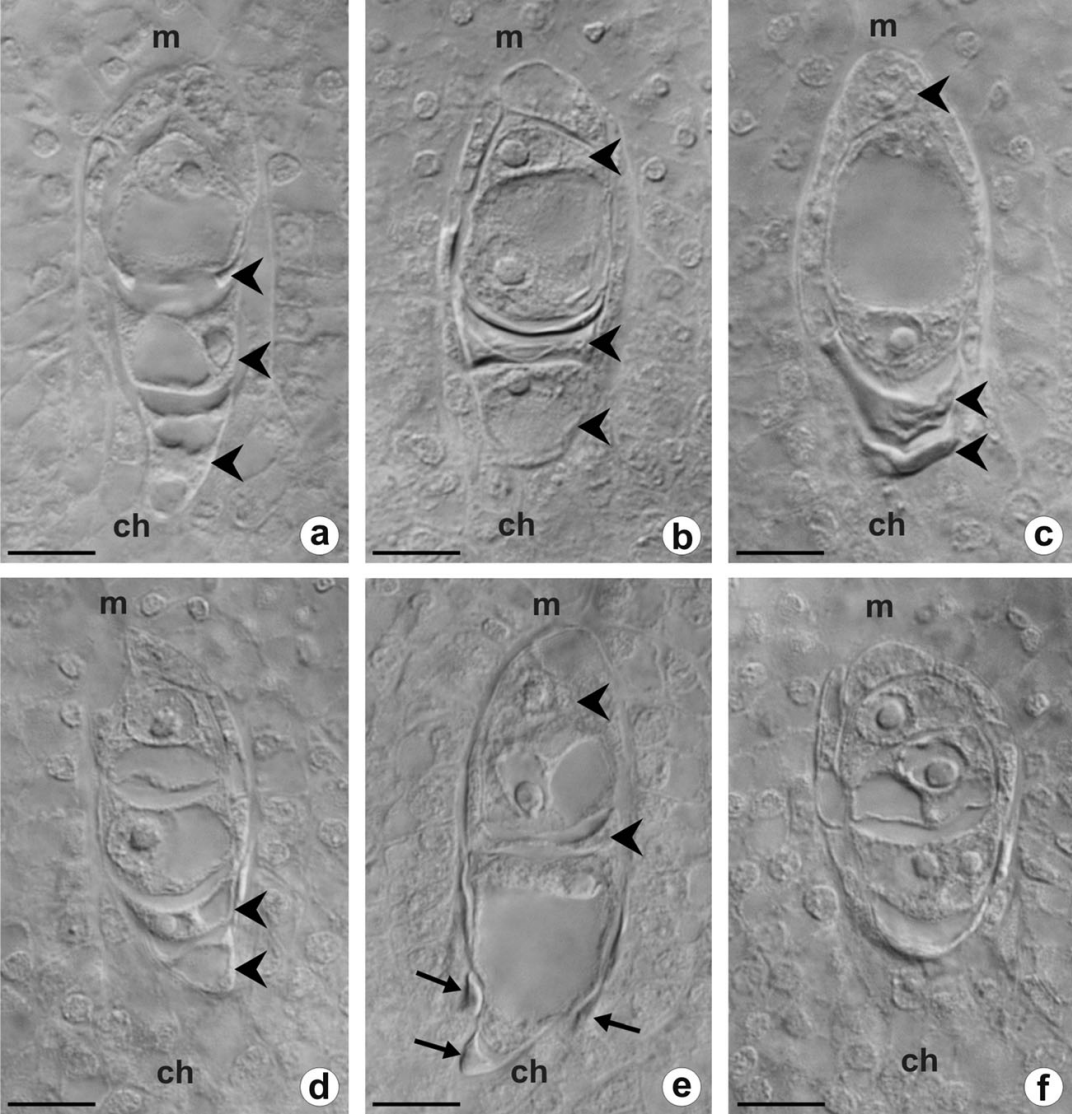
Megaspore tetrads of *T. linearisquameum* with various position of functional cell. Images were obtained from unstained, cleared flowers using Nomarski DIC optics. **a** Enlarged micropylar megaspore. **b, c** Increased submicropylar megaspores. **d** Megaspore tetrad with bigger and vacuolated micropylar and submicropylar cells. **e** Enlarged submicropylar and subchalazal megaspores; *arrows* show crushed cells of nucellus. **f** Three, instead of four, megaspores; two nuclei in chalazal megaspore. *Arrowheads* in all images indicate degenerating megaspores. *ch* chalazal pole, *m* micropylar pole. *Scale bars* = 10 μm

In the young ovules, when the integument begins to develop, a single archesporial cell differentiates in the hypodermal region of the nucellus. The archesporial cell develops directly into the MMC which undergoes a regular meiosis. After the first meiotic division, two dyad cells arise (Fig. 2a). The second meiotic division gives rise to a linear tetrad of haploid megaspores (Fig. 2b). Although the integmentary tapetum begins to differentiate at the tetrad stage, a fully formed layer of the integumentary tapetum is clearly visible at about the two-nucleate embryo sac stage (not shown). Usually, the three micropylar megaspores of the tetrad shrink and gradually degenerate, while the chalazal megaspore continues to develop into a FM (Fig. 2c). The FM greatly increases in size and then differentiates into a vacuolated embryo sac which undergoes three mitotic divisions without cytokinesis to form an eight-nucleate female gametophyte of *Polygonum* type. In *T. linearisquameum*, however, ovules from the same inflorescence may show developmental variants in which the FM is not the most chalazally located megaspore (Fig. 3a–e). In some ovules, the micropylar or submicropylar cell of the megaspore tetrad enlarges, while remaining megaspores degenerate (Fig. 3a–c). Such an increased megaspore has a conspicuous nucleus and highly vacuolated cytoplasm as in the cell of FM. In some linear tetrads, two megaspores may be distinctly increased in size and do not show signs of degeneration (Fig. 3d, e). Sporadically, because of disturbances in cytokinesis, three megaspores arise as a result of meiosis, but the chalazal cell is always two-nucleate (Fig. 3f).

**Fig. 4:**
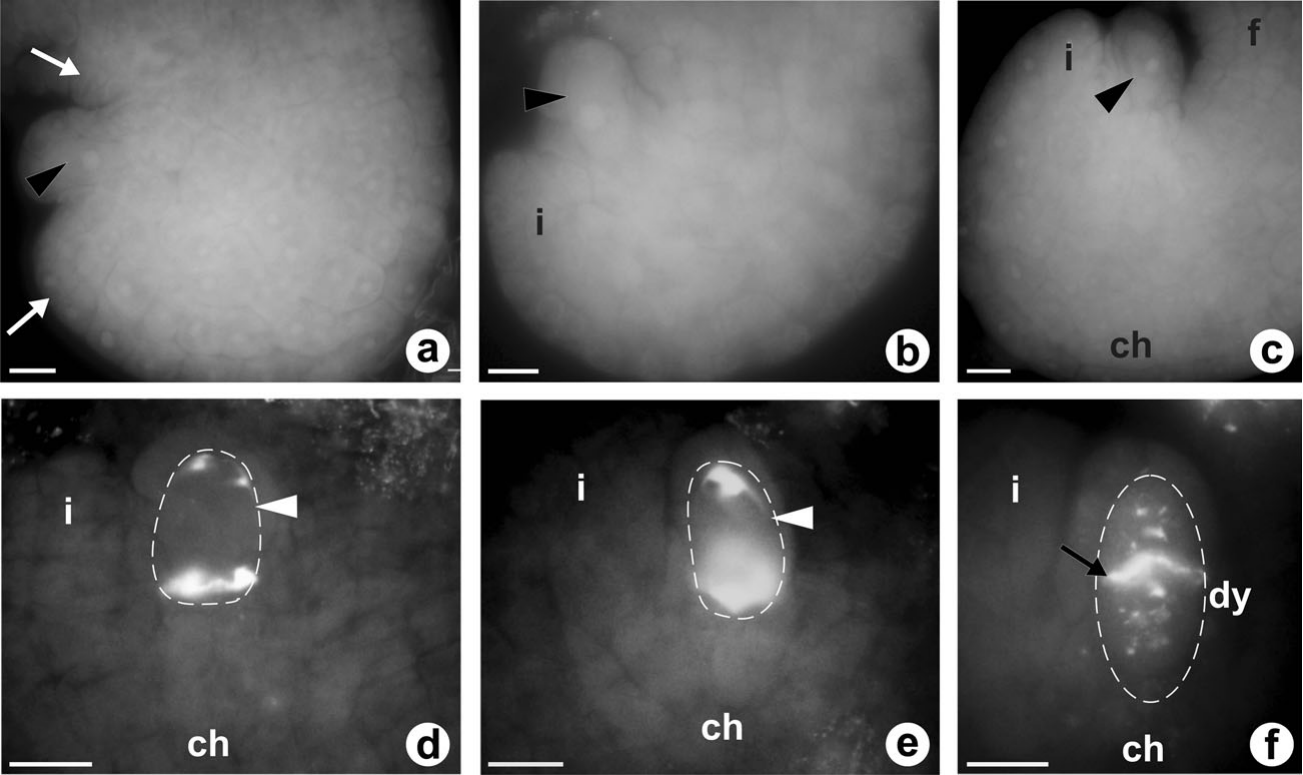
Callose localization in ovules of sexual *T. linearisquameum*. **a**–**c** Early consecutive developmental phases of anatropous ovule characterized by complete absence of callose; *arrows* show initiation of integument (*i*). *Arrowheads* point to archesporial cell, *ch* chalazal pole, *f* funicle. **d** Megaspore mother cell (*arrowhead*) with callose evident at the chalazal pole (*ch*); *i* integument. **e** Bipolar manner of callose deposition in the megaspore mother cell (*arrowhead*); *ch* chalazal pole, *i* integument. **f** Dyad of cells (*dy*) resulting from first meiotic division; *arrow* points to callose accumulation in transversal wall, *ch* chalazal pole, *i* integument. *Scale bars* = 10 μm

### Callose deposition during meiotic and diplosporous pathway of megaspores formation

**Fig. 5:**
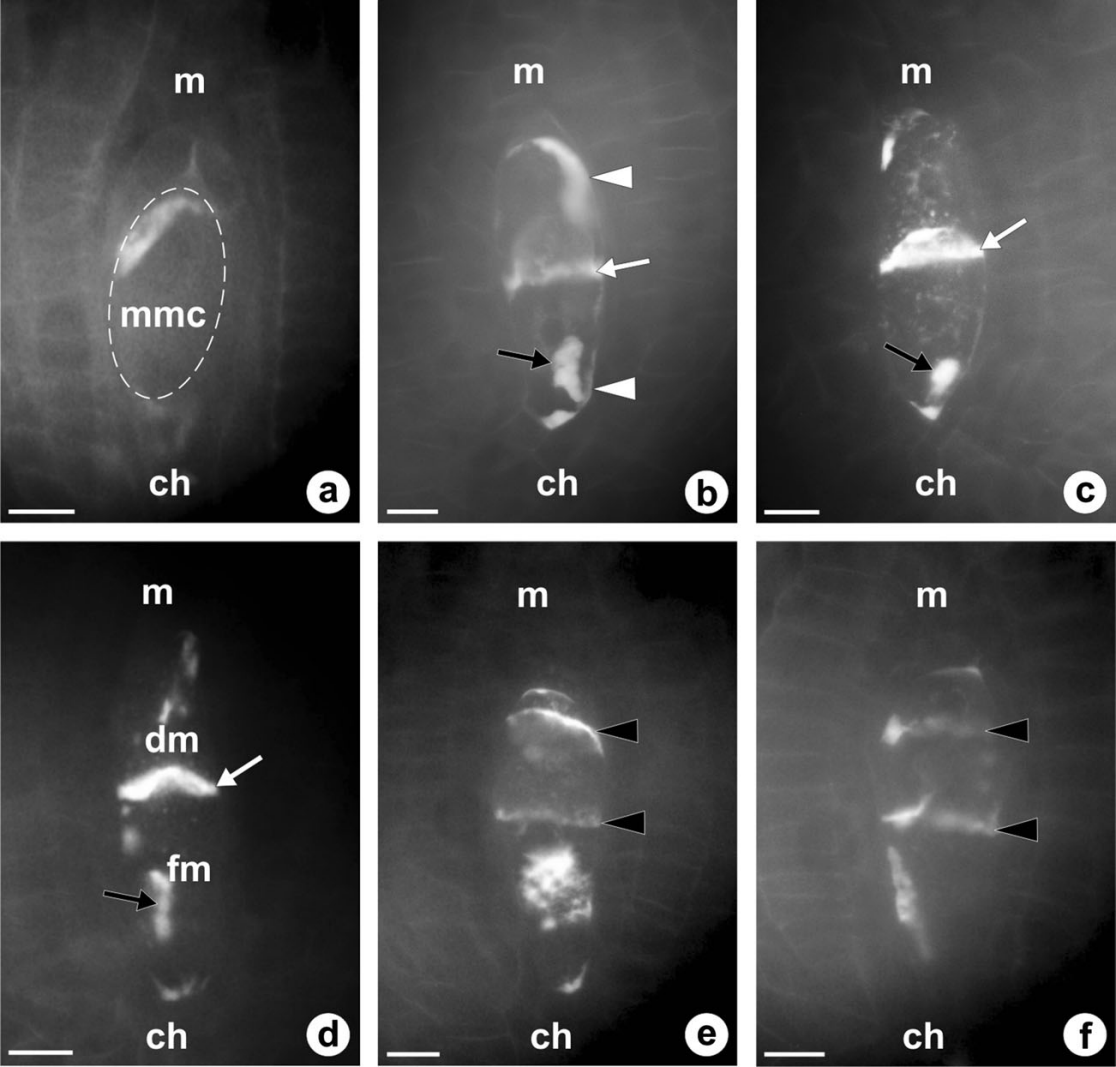
Callose localization in ovules of diplosporous *T. atricapillum*. **a** Megaspore mother cell (*mmc*) with callose evident at the micropylar pole (*m*); *ch* chalazal pole. **b** Early diplodyad; callose is visible both in the transversal wall (*white arrow*) and in the side walls of cells (*arrowheads*); *black arrow* shows the residues of degenerated nucellar cells. **c** Older diplodyad; transversal wall (*white arrow*) is the major site of callose deposition. *Black arrow* shows the residues of degenerated nucellar cells. **d** Functional megaspore (*fm*) with a visible callose accumulation (*white arrow*) at the micropylar pole. *Black arrow* shows the residues of degenerated nucellar cells, *dm* degenerating megaspore. **e**–**f** Callose deposition in transversal walls (*arrowheads*) of rarely occurred triads. *Scale bars* = 10 μm

**Fig. 6:**
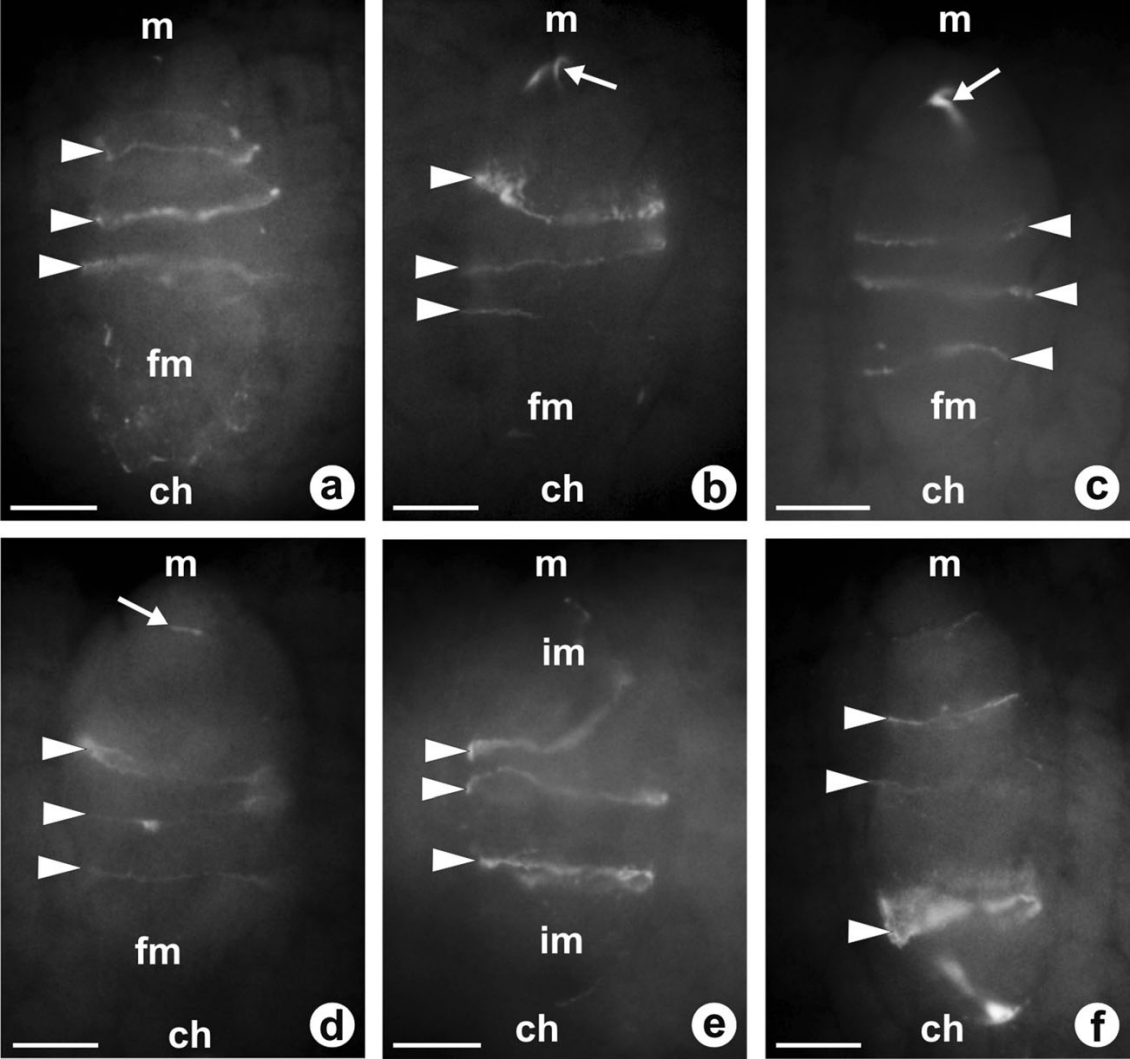
Callose distribution in tetrads of sexual *T. linearisquameum*. In all images transversal walls with callose denoted by *arrowheads; ch* chalazal pole, *m* micropylar pole. **a** Diffuse of callose fluorescence around differentiating chalazal functional megaspore (*fm*). **b**–**d** No callose in the wall of functional chalazal megaspore (*fm*); *arrows* indicate the presence of callose at the micropylar pole of the micropylar-most megaspore. **e** Lack of callose in the walls of increased micropylar and chalazal megaspores; *im* increased megaspore. **f** Callose accumulation around chalazal megaspore. *Scale bars* = 10 μm

Observations of the pattern of callose deposition in sexual *T. linearisquameum* and apomictic *T. atricapillum* show that callose is absent in somatic cells of the ovules as well as in the wall of the archesporial cell (Fig. 4a–c). In both examined dandelion species, DAB staining revealed that callose appears in the wall of megasporocyte at the early first meiotic prophase; however, the pattern of callose deposition is slightly different in the sexual and apomictic plants. In the ovules of *T. linearisquameum*, callose is initially clearly detectable at the chalazal pole and then it is also deposited at the micropylar pole of MMC (Fig. 4d, e). During a dyad stage, callose is principally located as a thick plate in the transverse wall separating each of the dyad cells, whereas it is not detectable in the side walls of the dyad (Fig. 4f). In apomictic *T. atricapillum*, callose is also deposited at both poles of MMC, but most callose is distinctly accumulated at the micropylar pole as crescent-like band (Fig. 5a). At the stage of diplodyad in an apomictic dandelion, callose is visible both in the wall between the diplodyad cells and in the side walls of the cells (Fig. 5b). Subsequently, callose becomes concentrated especially in the transversal wall (Fig. 5c). While the chalazal cell of diplodyad enlarges and becomes the FM, callose gradually disappears around the cell, but its accumulation is detected at the micropylar pole of the FM (Fig. 5d). The callose-containing walls are also characteristic for the cells of triads which have rarely been observed in the ovules of apomictic *T. atricapillum* (Fig. 5e, f). In the tetrads of *T. linearisquameum*, callose is present mainly in the transverse walls separating each of the megaspores; however, some variations in the intensity of fluorescence are noticeable in particular walls (Fig.6a–f). Usually, spotted fluorescence signals are additionally visible around the most chalazally situated and increased megaspore (Fig. 6a). This diffuse callose fluorescence indicates a gradual disappearance of callose in the wall of differentiating FM, and as a result, the wall of a selected megaspore is devoid of callose. Although in some tetrads, both chalazal and micropylar megaspore grow in size, no callose is detected around the chalazal cell, whereas callose is visible at the top of the micropylar megaspore (Fig. 6b–d). However, occasionally, callose is absent around both increased megaspores (Fig. 6e). Only sporadically, the amount of callose deposition does not decrease in the wall of the chalazal megaspore, and in these tetrads, a diminished callose deposition is noticed in the wall of the micropylar cell (Fig. 6f). It seems that some variations in the pattern of callose deposition in the tetrad reflect the variable positioning of the FM observed in the examined ovules of *T. linearisquameum*.

## Discussion

It is well known that callose occurs in various plant tissues as a component of specialized cell walls at certain stages of growth and that its deposition can be also induced locally by physiological stress, wounding, and pathogen infection (Stone and Clarke 1992). Moreover, callose synthesis is an initial symptom of ovule abortion as well as of embryo senescence (Vishnyakova 1991; Sun et al. 2004; Teng et al. 2006). Although in the ovules of the majority of flowering plants callose is a cytological marker of MMC wall and megaspores walls, its role in the megasporogenesis, as well as in the selection of FM, is not fully understood (Tucker et al. 2001; Bicknell and Koltunow 2004; Tucker and Koltunow 2009; Drews and Koltunow 2011). Additionally, the pattern of callose deposition and degradation during angiosperms megasporogenesis still remains relatively poorly documented (Lersten 2004).

To date, research data on callose localization in *Taraxacum* ovules are missing, and this report is the first documentation of callose events during megasporogenesis in dandelions. We demonstrated the deposition of callose in the walls of cells undergoing megasporogenesis both in sexually reproducing *T. linearisquameum* and in diplosporous *T. atricapillum*. In the ovules of diploid *T. linearisquameum*, the pattern of callose deposition is similar to the pattern previously reported in monosporic species that form the *Polygonum* type of embryo sac (Rodkiewicz 1970). In triploid *T. atricapillum*, unreduced megaspores are a result of the meiotic diplospory of *Taraxacum* type, and during the formation of megaspores, a bipolar deposition of callose is also characteristic for the wall of MMC, but in contrast to the sexual dandelion, callose accumulates mostly at the micropylar pole of the cell. Previously, it was suggested, basing on the studies of diplosporous grasses, that the absence of callose during megasporogenesis may be characteristic of most types of diplospory (Carman et al. 1991; Leblanc et al. 1995; Peel et al. 1997). Our results, revealing the presence of callose in the walls of MMCs of apomictic dandelion, are distinct from the earlier reports for diplosporous *Tripsacum* (Leblanc et al. 1995; Peel et al. 1997). In this grass, unreduced embryo sacs are produced by diplospory mainly of the *Antennaria* type, but the *Taraxacum* type of diplospory has also been observed. However, regardless of the mode of diplospory, callose was absent in the wall of MMCs (Leblanc et al. 1995; Peel et al. 1997). On the other hand, a total absence or a distinct restriction of callose to the micropylar pole of the MMC was detected in *E. rectisetus* that commonly undergoes diplospory of the *Taraxacum* type (Carman et al. 1991). Similar accumulation of callose, mainly at the micropylar pole, is detected in the cell walls of megasporocytes in *T. atricapillum*. It therefore seems that in diplosporous species, the pattern of callose deposition in the wall of MMC is related to the type of diplospory. In the case of *Antennaria* type, the wall of MMC is devoid of callose, whereas the limitation of callose to the wall of MMC closest to the micropyle may be correlated with abnormal asynaptic meiosis and may indicate diplospory of the *Taraxacum* type. Callose was also detected around the megasporocytes as well as in the transversal walls between megaspores of apomictic monosomic addition line of *B. corolliflora* exhibiting diplospory of *Allium odorum* type in which a normal meiosis in the MMC is preceded by a premeiotic endomitosis (Shen et al. 2006). However, the absence of callose in the wall of megasporocytes in the species with the tetrasporic type of megagametophyte formation does not interfere with meiosis, and normal meiotic divisions occur in the absence of callose deposition (Rodkiewicz 1970).

In most flowering plants, the differentiation of the FM is crucial for female gametogenesis, but so far the genetic basis and molecular mechanisms that regulate the selection and fate of megaspores remain unknown. However, recent findings provided new data on the factors influencing the megaspore’s fate. For example, in *Lactuca sativa*, the relationship between changes in dynamic calcium (Ca^2+^) concentration and megaspore degeneration was stated (Qiu et al 2008). Furthermore, it was shown that a chalazal-located sporophytic cytokinin signal as well as a classical arabinogalactan protein AGP18 have a significant role in the specification of the *Arabidopsis thaliana* FM (Cheng et al. 2013; Demesa-Arévalo and Vielle-Calzada 2013). It is also established that callose deposition has been correlated with the selection of the FM and the main function of callose is to suppress non-functional megaspores by isolation (Webb and Gunning 1990; Russell 1979). However, it remains unclear whether the presence of callose or its deposition in a particular pattern around the megaspores influences their development (Tucker and Koltunow 2009). Thus, a comparison of callose deposition patterns in the ovules of sexual and apomictic plants is valid, especially in light of recent studies regarding the molecular mechanisms controlling the apomictic mode of reproduction which indicate that apomixis is an altered form of sexuality with respect to spatial and temporal deregulation of the expression of genes involved in female sexual reproduction (Barcaccia and Albertini 2013 and references therein). Therefore, deeper knowledge of the mechanisms that regulate reproductive processes in plants is required, including detailed descriptive studies.

In conclusion, the results of our comparative studies concerning callose events in the ovules of sexual and apomictic dandelions revealed that (i) somatic tissues of young dandelions ovules are free of callose; (ii) callose is present in the wall of megasporocytes both in diplosporous *T. atricapillum* and sexual *T. linearisquameum*; (iii) in both examined species, callose is deposited in a bipolar manner in the MMC; however, in a diplosporous dandelion, callose predominated at the micropylar pole of MMC; (iv) callose is present in the transversal walls separating megaspores within tetrads of a sexual dandelion; (v) callose accumulates also in the wall between cells of diplodyad in apomictic *T. atricapillum;* and (vi) callose disappears in the wall of FM during both the meiotic and diplosporous pathway of development.
